# The natural history of IgE mediated wheat allergy in children with dominant gastrointestinal symptoms

**DOI:** 10.1186/1710-1492-10-12

**Published:** 2014-02-26

**Authors:** Grażyna Czaja-Bulsa, Michał Bulsa

**Affiliations:** 1 Pediatrics and Pediatric Nursery Unit of the Pomeranian Medical University in Szczecin, Szczecin, Poland; 2 Division of Paediatrics, Gastroenterology and Rheumatology of the "Zdroje" Hospital in Szczecin, Szczecin, Poland

**Keywords:** Food allergy, Food tolerance, Wheat allergy, Specific immunoglobulin E, Children

## Abstract

**Background:**

Wheat is one of the most common food allergens in children. The purpose of this study is to define the natural course of wheat allergy in children with dominant gastrointestinal symptoms and identify factors that help predict development of tolerance.

**Methods:**

The prospective analysis covered 50 children with positive food challenge results (DBPCFC) and positive wheat IgE test result. Resolution of wheat allergy was determined on the basis of food challenge results (open challenge). The impact of each of the studied factors on the age when tolerance developed was assessed by means of the Cox proportional hazard regression model.

**Results:**

The median age of tolerance development was 69.5 months (37-192 mo.). The rates of resolution were 20% by the age of 4 years, 52% by the age of 8 years, and 66% by 12 years, and 76% by 18 years. The median age of the tolerance development in children with peak wheat IgE level below10 kU/L was 41.4 months, with peak wheat IgE from 10 to 19.9 kU/L was 44.5 months, with peak IgE from 20 to 49.9 kU/L – 84,9 months and with peak IgE ≥ 50 kU/L – 190.5 months. The median of the age when the highest levels of IgE for wheat were reached was 33 months (2-52 mo.) in children with resolved wheat allergy and 67 months (36-178 mo.) in children with persistent allergy (p = .001).

**Conclusions:**

1. The majority of children with wheat allergy can tolerate wheat by adolescence. 2. The age when tolerance to wheat developed depended on the level and the age of reaching the highest levels of specific IgE for wheat. The higher the values of the above parameters, the older a child was when they developed tolerance to wheat.

## Introduction

Wheat is one of the most commonly consumed types of grain in Europe and in America. As one of the gluten cereals it is responsible for numerous diseases which are called gluten-related disorders [1]. According to the most recent classification they are divided into three main groups: allergic, autoimmunological (celiac disease, dermatitis herpetiformis) and possibly immune-mediated (gluten sensitivity) [1]. The frequency of the former two is about 1% each, while the third one may affect 6% of population [2-5]. Although wheat is one of the most common food allergens in children, the natural history of the IgE-dependent allergy to wheat (WA) has been rarely discussed in the literature [6-10].

The purpose of this study is to define the natural course of WA in a group of children with predominant gastrological symptoms and to identify factors that help predict the development of tolerance.

## Methods

**Figure 1:**
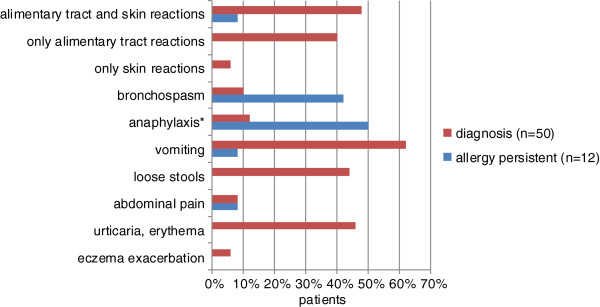
Type of adverse reactions to wheat during the first 18 years of life.

The prospective analysis covered 50 children with WA who were the outpatients of the Gastrology Department and the Gastrology Outpatient Clinic Pomeranian Medical University in Szczecin and the "Zdroje" Hospital in Szczecin. They remained under the care of an attending physician (an allergologist and gastroenterologist) throughout the whole observation period. All the patients reported gastrointestinal symptoms (vomiting, loose stools, abdominal pains) and/or wheat allergy suspicion. All the patients at initial diagnosis had positive food challenge results (with symptoms occurring within 2 hours after wheat consumption) and positive SPT as well as the levels of sIgE for wheat (wsIgE) higher than 0.7 kU/L (Figure 1). The study was conducted from January 1990 to May 2012.

The inclusion criteria were: diagnosed WA and consent of participants and their guardians.

The criterions to exclude a child from the observation were other chronic diseases*.* Only 4 children were lost to follow-up: three children who visited the clinic only once and one child with ulcerative colitis. Individual observations were terminated when a patient’s tolerance to wheat (WT) had developed or when the patient turned 18.

The medical interview included questions about a patient’s age, gender, family history of allergies, coexisting allergic diseases as well as other food allergies. At initial diagnosis and at the every 1-2 years follow-up visit examinations were performed including: SPT, wheat- and gluten sIgE (wsIgE, gsIgE) tests as well as wheat challenge tests. The analysis covered the results of tests taken at the time of diagnosis (wsIgE_1_, gsIgE_1_) and after 1-2 years of the elimination diet (wsIgE_2_, gsIgE_2_); the highest test results (wsIgE_max_, gsIgE_max_); the results obtained during tolerance (wsIgE_3_, gsIgE_3_). In order to investigate the dependence between the sIgE_max_ level and the age when the tolerance to wheat developed the patients were divided into 4 groups: <10 kU/L (n = 7), 10-19.9 kU/L (n = 13), 20-49.9 kU/L (n = 25) and ≥50 kU/L (n = 5).

SPTs were performed by means of commercial solutions of food allergens manufactured by Allergopharma (Germany). Specific IgE in serum were determined by means the FEIA method and performed with the aid of ImmunoCAP System (Pharmacia & Upjohn Diagnostics AB, Uppsala, Sweden).

Food challenge tests (FC) were performed by means of the open food challenge (OFC) or a double-blind placebo-controlled food challenge (DBPCFC). The first challenge test was performed as the OFC, the second - always as the DBPCFC followed by the OFCs. Food challenges were not repeated in children with severe anaphylactic reaction requiring epinephrine administration. The scheme was based on the contemporary recommendations of the Section of Allergies and Food Intolerance of the Polish Society of Paediatric Gastrology which followed the work of Bock et al. [11,12]. The OFC began with the application of a drop of wheat groats - semolina (10% wheat cooked in water) on the upper lip. Thereafter wheat was given orally in increasing amounts in 15-minute intervals starting from a dose of 50 mg to reach the total of 18-20 g (3.24-3.6 g of wheat protein). If there was no reaction to the food challenge, wheat was administered daily (>20 g of wheat) at home for 7 days by the open method to ensure that there were no other adverse symptoms (i.e. to rule out a co-existing non-atopic allergy). When no adverse effects appeared, WT was diagnosed. If the adverse effects had been observed, the food challenge was abandoned and WA was diagnosed. We diagnosed persistent wheat allergy in those children in whom the symptoms continued in the 18th year of age or when the observation came to an end.

To perform DBPCFC the authors used mainly liophilised food manufactured by Apipol-Farma (in capsules) and native foods in highly hydrolysed casein (Nutramigen), or, in other native tolerated foods. As placebo they used maltodextrin (117 mg) and colloidal silica (13 mg) in capsules or other native tolerated foods.

In children whose WA had been diagnosed in infancy the SPT and sIgE were assayed at the diagnosis and the food challenge was performed after the 10th month of life. In Poland it is recommended to introduce wheat in unlimited quantities into the infant diet at that age.

Asthma, atopic dermatitis and allergic rhinitis were diagnosed by an attending physician (an allergologist). An IgE-dependent allergy to other foods was diagnosed in children who had positive food challenge (OFC or DBPCFC, depending on age), and had positive SPT and/or sIgE. Anaphylaxis was defined as a severe reaction involving two or more body systems or the lower respiratory tract.

The data were characterised by the median, the minimum and maximum values. The development of tolerance depending on the examined factors was assessed by means of the Pearson Chi^2^ test. The Mann-Whitney test was used to compare wheat IgE levels between the patients with persistent allergy and the patients who had developed tolerance. The impact of each of the studied factors on the age of tolerance development was assessed by means of the Cox proportional hazard regression model and presented with the Kaplan-Meier curves, while the significance of the curves was measured with the log-rank test. The correlation between the selected variables was evaluated with the Spearman rank correlation. The tool used for the statistical analysis was STATA 11, License No 30110532736.

The study was conducted in compliance with the Declaration of Helsinki and following the consent by the Ethical Committee of the Pomeranian Medical University in Szczecin (BN-001/107/90). Initially, the study was financed from the grant No4 PO5E 086 14 of the State Committee for Scientific Research followed by funding from the clinic in-house research budget.

## Results

A natural process of WA was analysed in 50 children - 32 boys and 18 girls from the median age of 6 months (2-8 mo.) to 216 months (36 - 216 mo.) (Table 1).

**Table 1:** Demographic characteristics of the study patients

**Characteristics**	**No. (%) of patients; n = 50**
Sex: male/female	32 (64)/18 (36)
Other atopic diseases*	
Eczema	39 (78)
Asthma	24 (48)
Allergic rhinitis	17 (34)
Eosinophyllic gastrointestinal disease*	6 (12)
Eosinophilic oesophagitis	5 (10)
Other food allergies*	
Milk	40 (80)
White egg	36 (72)
Soy	12 (24)
Fish	14 (28)
Peanut	25 (50)
Tree nuts	13 (26)
Number of food allergens:*	Median – 4; range: 3-7
3	4 (8)
4	33 (66)
≥5	13 (26)
Family history of atopy	50 (100)
1 parents	9 (18)
Both parents	41 (82)
Siblings	24 (62)

*throughout the whole observation period.

The median age of WA diagnosis was 13 months (2-22 mo.). It did not differ significantly between the children who developed WT and those who did not*.* In 32% of the WA children the disease manifested itself as early as in infancy, the number including 6% of children who developed symptoms while being breastfed. The types of adverse reaction to wheat during diagnosis are presented in Figure 1. They were similar to the reactions reported before diagnosis by the patients’ parents. The parents reported several of them more often than it was in case of the FC: skin reactions (58% vs. 54%), bronchospasm (14% vs. 10%) and abdominal pain (18% vs. 8%)

In group of the WA children 302 tests for wsIgE were conducted. At the WA diagnosis median wsIgE_1_ was 8.42 kU/L (2.2-39.3 kU/L). It did not differ significantly between the children who developed WT and those who did not. The median of wsIgE_2_ was significantly higher (p = .048) in children who did not develop WT in comparison to those who did: 16.0 kU/L (3.2-59.3 kU/L) vs 9.12 kU/L (1.8-63.4 kU/L), respectively. The median of wsIgE_max_ was also significantly higher (p = .04) in children with persistent allergy 62.7 kU/L (36.3-72.4 kU/L) than in those with resolved allergy 13.5 kU/L (7.9-59.3 kU/L). Disregarding the age, all the children who did not develop WT demonstrated higher values of wsIgE than those who did (Table 2). In the group of the WT children the median of wsIgE_3_ was 3.0 kU/L (0.35-23.9 kU/L).

**Table 2:** Median wheat IgE levels in patients with persistent and resolved wheat allergy

**Age (years)**	**Wheat specific IgE (kU/L)**		p value*
	**Outgrown**	**Persistent**	
	**n = 216**	**n = 86**	
0-2	9	19	.04
2-4	10	27	.03
4-6	7	49	.03
6-8	6	46	.04
8-10	6	42	.04
10-12	5	36	.05
12-14	4	35	.07
14-16	4	33	.14
16-18	4	30	.36

*Mann-Whitney test.

At the WT diagnosis the gluten specific IgE > 0.35 kU/L was observed in 68% of children, while the concentration at 0.7 kU/L - in 46%, with the same frequency in those children who developed and did not develop the WT. The value of gsIgE_max_ was 5 kU/L (0.35-46.9 kU/L). The medians gsIgE_1_, gsIgE_2_ and gsIgE_max_ did not differ significantly between the compared groups of children.

The factor that significantly distinguished both groups was the age at which the children reached wsIgE_max_. The children with developed WT reached it earlier than those who did not: 33 months (2-52 mo.) vs. 67 months (36-178 mo.) (p = .001). Such dependence was not observed in case of gsIgE_max_.

76% of children developed WT during the observation period. The median of tolerance development age was 69.5 months (37-192 mo.). The rates of resolution were 20% by the age of 4 years, 52% by the age of 8 years, and 66% by 12 years, and 76% by 18 years.

The median of the tolerance development age in children with wsIgE_max_ below 10 kU/L was 41.4 months, with wsIgE_max_ from 10 to19.9 kU/L – 44.5 months, with wsIgE_max_ from 20 to 49.9 kU/L – 84.9 months and with wsIgE_max_ ≥ 50 kU/L – 190.5 months. During the observation period WT developed in 80% of children with wsIgE_max_ below 50 kU/L and in 40% of children with wsIgE_max_ ≥50 kU/L.

**Figure 2:**
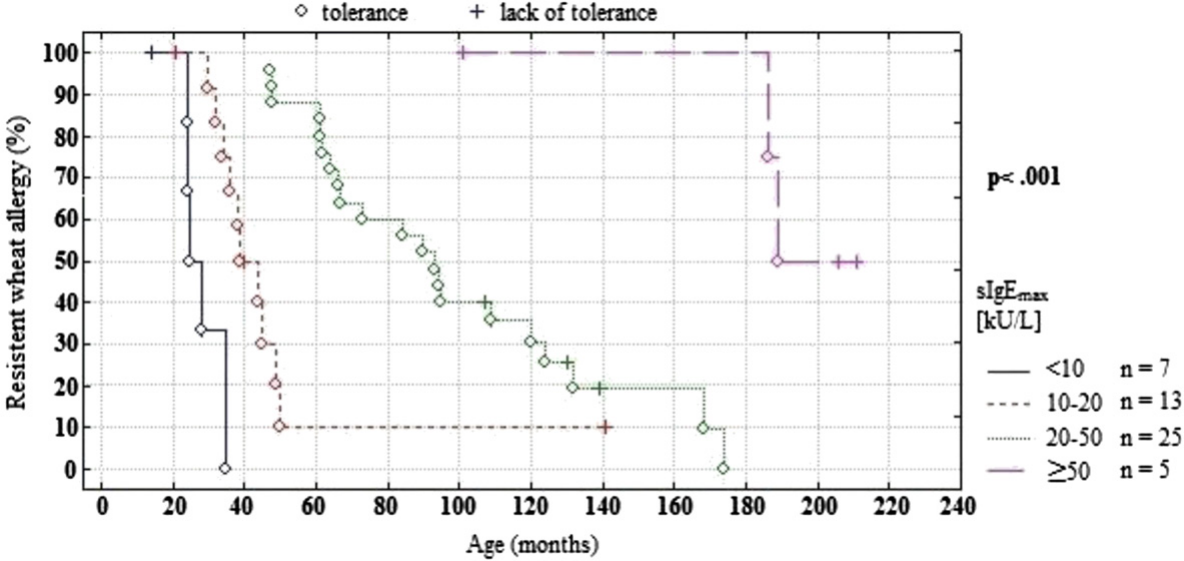
Relationship of peak wheat IgE level to persistence of wheat allergy during the first 18 years of life.

The study revealed that the age when WT developed depended on the level of wsIgE_2_ as well as on the level and the age of reaching wsIgE_max_ (Table 3, Figure 2). The higher the values of the above parameters were, the older a child was when they developed WT. Other factors such as gender, family history of atopy, the age at the WA diagnosis, the type of the adverse reaction to food challenge, the number and kind of other food allergens, coexistence of another allergic disease, the level of wsIgE_1_, gsIgE_1_, gsIgE_2_ and gsIgE_max_ did not have any significant effect on the age when WT developed.

**Table 3:** The impact of chosen factors on the tolerance development age in children with IgE-dependent allergy to wheat

**Parameter**	**n**	**Cox proportional hazard regression model**	
		**r**	**p**
Wheat IgE_1_ [kU/L]	50	- .023	.273
Wheat IgE_2_ [kU/L]	50	- .052	.001
Wheat IgE_max_ [kU/L]	50	- .113	.001
Gluten IgE_max_ [kU/L]	34	- .019	.149
Age of wheat allergy diagnosis [mo.]	50	.054	.089
Age of wheat sIgE_max_ [mo.]	50	- .076	.001
Number of food allergens	50	.309	.097
Coexistence of another allergic disease	50	.240	.074

IgE_1_: diagnosis, IgE_2_: 1-2 years after diagnosis; r – correlation coefficient.

**Figure 3:**
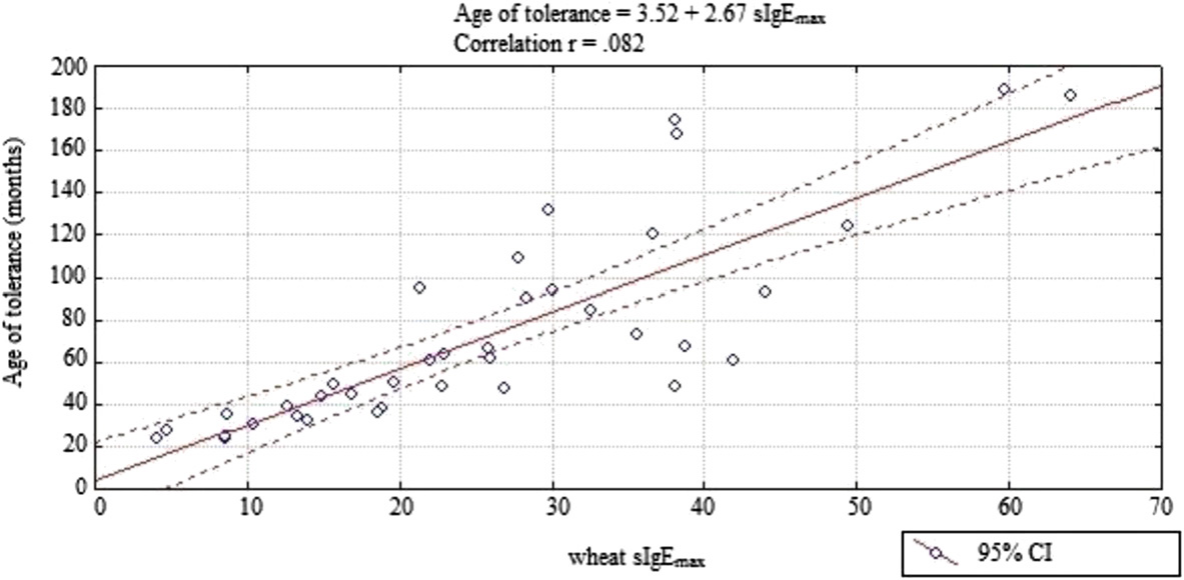
The curve of the correlation of the peak wheat sIgE level with the wheat tolerance development age.

In children who developed WT a significant correlation was observed between the age of tolerance development and the level of wsIgE_max_ (r = .82, p < .001) (Figure 3).

## Discussion

The paper presents the natural history of WA in children with predominant gastrointestinal symptoms. They affected 88% of patients and mainly included vomiting (62%) and loose stools (44%). Abdominal pain was rarely reported (8%), which results from the patients’ young age. There have been no similar descriptions in the literature. Few publications presenting the WA course dealt mainly with children with dominating skin and respiratory symptoms as well as with anaphylaxis [6-10].

Similar to other authors, we found that children with WA have high atopic potential. They come from families with history of atopy [6,7]. Over the years most of them develop other atopic diseases, most frequently atopic dermatitis (78%), allergic rhinitis (64%) and asthma (48%) [6,7,9]. They suffer from food allergies, particularly to milk (80%), egg white (72%) and peanuts (50%), but also to other nuts, fish and soya [6,7].

In the study group the median of WA diagnosis age was 13 months (2-22 mo.). In one third of the children the WA signs appeared in infancy and in 6% during breastfeeding. In all the children other food allergies had been diagnosed before wheat. As in other studies the median of the food allergy diagnosis age was 3 months (1-8 mo.) [7,9]. Some authors quote higher age of the WA diagnosis [6,13]. Most patients (76%) had developed WT before reaching maturity. The WT age was 5.5 years (3-16 y*.).*

According to other authors the IgE-dependent allergy to milk or hen egg requires similar periods of elimination diet. In their studies 60% to 80% of patients reached tolerance also at the age of 8-12 years [14-16]. Worse prognosis was reported for children with IgE-dependent allergy to peanuts and other nuts as well as to sesame seed. Only 10-20% of them developed tolerance, while in the majority of cases the allergy continued into adulthood [17].

The risk of continued WA was high in those diagnosed children who, despite the elimination diet, had been demonstrating rising wsIgE for several years. The median of age in which those children reached maximum concentration of wsIgE was 5.5 years (3-14.5 yr).

In children who developed WT, after 1-2 years of diet the median of wsIgE did not differ significantly from the level at diagnosis. Those children reached maximum concentrations of wsIgE as early as at 2.7 years of age (2 mo.-4.5 yr.). Then the wsIgE values fell, but in some children they did not return to the level below 0.7 kU/L.

An important factor that modified the WT age was the level of wsIgE_max_. There was a significant difference between the children with wsIgE_max_ below 20 kU/L, at 20-50 kU/L and over 50 kU/L. Their tolerance age medians were, respectively: 3.5 years, 7 years and 16 years. There was no relevant difference between the children with wsIgE below 10 kU/L and at 10-20 kU/L.

Having determined both the age when wsIgE_max_ was reached and its actual value, we can predict the further course of the disease [6,18,19]. Reaching wsIgE_max_ in older age is an important factor increasing the risk that the allergy will continue into adulthood. Such children demonstrate fast rising wsIgE in the initial period of the diet and maintain its high levels in the subsequent years (often >50 kU/L). The consumption of wheat leads to the signs of anaphylaxis. In our study the specific IgE for gluten did not display such dependence and therefore we believe that it is unlikely to provide any benefit in clinical practice and it is not important from the clinical point of view.

What was characteristic of the children with WT were persistently high levels of wsIgE – their median reached 3 kU/L (0.35-23.9 kU/L) [6,10]. There could be several reasons for this. The first reason are cross reactions among the water- and salt soluble wheat grain and pollen proteins responsible for the false positive results [20]. The second reason is insufficient knowledge about the wheat grain allergens. Solutions used for SPTs or for determining sIgE for wheat do not contain all the wheat grain allergens. The example is rω-5 gliadin which must be determined independently [10]. And thirdly, it is likely that, as in the case of allergies to milk or hen egg, different epitopes are recognised by antibodies of the patients who will develop tolerance and of the ones whose allergy will continue [21]. Battais et al. proved that IgE of those suffering from wheat dependent exercise induced anaphylaxis (WDEIA), anaphylaxis and urticaria detected sequential epitopes in the repetitive domain of gliadins, whereas the IgE of atopic dermatitis patients probably recognized conformational epitopes [22].

In milk, hen egg and peanuts allergies the reduction in SPT and the reduction of sIgE concentration below a cut-off are popular indicators of the high likelihood of losing clinical reactivity by allergic people [14,15,18,23-25]. In such cases the assessment of the sIgE values in subsequent years of elimination diet is a useful tool to prognosticate tolerance.

As other researchers have found, the volume of sIgE concentration is not a useful factor of the clinical reactivity to wheat [10,26]. Using the CAP system, the positive predictive value of wheat sIgE remained less than 75% [26]. In recent years a new wheat allergen, the recombinant rω-5-gliadin (Tri a 19), has been discovered in patients with anaphylaxis and with WDEIA. High concentrations of IgE for the recombinant rω-5-gliadin correlate with the severity of response as well as with the wheat volume in a food challenge test., which gives hope that the cut-off value can be determined giving the maximum efficiency of the rω-5-gliadin test for the positive wheat challenge [8,10,27-29]. In other forms of WA the rω-5 gliadin-specific IgE is less frequent – it is observed in just 30% of patients. Moreover, in these groups of patients there is no correlation between the concentration of the rω-5 gliadin-specific IgE and food challenge test results [30].

In conclusion, children with WA demonstrating predominant gastrointestinal symptoms develop WT in childhood - ^2^/_3_ of them by the age of 10. In the majority of cases it is not related to normalisation of wsIgE, which can remain high. The level and age of peak level of wsIgE are an important factor enabling us to predict when WT will appear. Coexisting allergic diseases and allergies to other foods are not the factors of negative prognosis.

## Abbreviations

WA: The IgE-dependent allergy to wheat; SPT: Skin prick tests; wsIgE: SIgE for wheat (wsIgE); WT: Tolerance to wheat (WT); wsIgE: Wheat sIgE; gsIgE: Gluten sIgE; wsIgE1: Wheat sIgE at the time of diagnosis; gsIgE1: Gluten sIgE at the time of diagnosis; wsIgE2: Wheat sIgE after 1-2 years of the elimination diet; gsIgE2: Gluten sIgE after 1-2 years of the elimination diet; wsIgEmax: The highest wheat sIgE; gsIgEmax: The highest gluten sIgE; wsIgE3: Wheat sIgE at the time of tolerance; gsIgE3: Gluten sIgE at the time of tolerance; OFC: Open food challenge; DBPCFC: Double-blind placebo-controlled food challenge; WDEIA: Wheat dependent exercise induced anaphylaxis.

## Competing interests

The authors declare that they have no competing interests.

## Authors’ contributions

Both authors read and approved the final manuscript.
